# Variation in the Prevalence of Domestic Violence between Neighboring Areas

**DOI:** 10.1155/2014/721951

**Published:** 2014-07-06

**Authors:** Sedigheh Nouhjah, Seyed Mahmood Latifi

**Affiliations:** Health Research Institute, Diabetes Research Centre, Jundishapur University of Medical Sciences, Ahvaz, Iran

## Abstract

Domestic violence against women is an important health issue, but few studies have focused on city of residence and ethnic differences. To estimate the prevalence of various forms of domestic violence and certain related factors, with a specific focus on city of residence and ethnicity, we studied 1820 married women attending public health centers in 4 large cities in Khuzestan Province, southwestern Islamic Republic of Iran. We used an interviewer-administered questionnaire for data collection. The prevalence of some forms of lifetime domestic violence against women was 47.3%. The prevalence of physical, psychological, and any form of lifetime violence was the highest in Dezful (25.7%, 54.8%, and 57.7%, resp.). For sexual violence, the highest prevalence was reported in Ahvaz (17.7%). The highest prevalence of physical and sexual violence during any point of life was reported by Arab women (25.1% and 16.7%). The experience of all forms of violence was significantly associated with city of residence. Results of regression logistic analysis revealed that all of the forms of violence except psychological violence were statistically significantly associated with ethnicity (*P* < 0.05).

## 1. Introduction

Domestic violence, the most common type of violence against women [1, 2], is well recognized as a serious risk factor for many physical, mental, and reproductive health consequences, as well as being a violation of human rights [2–5]. The immediate and long-term health outcomes that have been linked to violence have been indicated in many studies; these include injury, unwanted pregnancy, abortion, sexually transmitted infections, posttraumatic stress disorder, and depression [6–8].

Violence against women, in its various forms, is endemic in communities and countries around the world, cutting across class, race, age, and religious and national boundaries [2]. In 48 population-based surveys from around the world, between 10% and 69% of women reported being physically assaulted by an intimate male partner at some point in their lives [9, 10].

Estimates of the prevalence of domestic violence within the Islamic Republic of Iran vary widely, from 10% to 55%, between studies in various forms of violence and using different methodologies [11–13].

As domestic violence against women becomes more recognized and discussed, questions are being raised concerning the magnitude of the problem in different settings and the causes, consequences, and related risk factors [2]. Widely differing prevalence rates across the world suggest that social and cultural contexts can play a major role in the perception of domestic violence against women [14]. Little is known about the related context, cultural, and social factors involved. Violence against women and related issues are unreported in many Iranian ethnic groups and cities.

There are 8 ethnic groups Baluch, Torkman, Talesh, Fars (Persian), Kord, Arab, Lur, and Iranian Arab in the Islamic Republic of Iran [15]. Three of these, Fars, Arab, and LurBakhtiary, comprise the main ethnic groups in Khuzestan (the overall majority of Iranian Arabs are resident in Khuzestan Province). The people of the Fars ethnic group are resident in Dezful, Ahvaz, and Abadan and the people of the Arab ethnic group in Ahvaz and Abadan. Almost all of the Lur ethnic group are resident in Andimesk.

The aim of this study was to estimate the prevalence of various forms of intimate partner violence and some related factors in 4 cities of Khuzestan and to make a comparison of the results, with a specific focus on city of residence and ethnicity.

## 2. Methods

In this cross-sectional study, 1820 married women who were attending public health centres in 4 largest cities of Khuzestan (one of the largest provinces in the south west of the Islamic Republic of Iran) were studied.

Four large cities, Ahvaz, Abadan, Dezful, and Andimesk, were selected for this study. These cities were selected as they are largely representative of the province. Ahvaz is the capital of the province and has a number of different ethnic groups; the others are also large cities with different cultural and ethnic groups. The population of these cities is about 1 million, 200 000, 400 000, and 200 000, respectively. The study was carried out from January 2007 to March 2008.

Since the prevalence of domestic violence in Khuzestan had not been previously estimated, we calculated the prevalence based on previous studies in other parts of the country [11, 16]. The required sample size was estimated using a World Health Organization manual [17]: 20% prevalence rate to a confidence level of 95% and 0.05 precision gave sample sizes of 500, 280, 480, and 200 women in Ahvaz, Andimesk, Dezful, and Abadan, respectively. We increased the sample size to 1820 to cover sampling error.

Convenience sampling was used for data collection. The interviewer attended the selected health center for 4 days a week. Every woman seeking family health services (contraception, routine care for children) during the study period was asked to participate in the study. Participants were assured of the confidentiality of their responses. Overall 1865 women in 4 cities were selected, but 45 women refused to participate in the research, a refusal rate of about 2.5%. A private room in the health center was available for conducting the interviews and completing the questionnaires. The unique file number for each participant was checked to ensure no woman was interviewed more than once.

We used an interviewer-administered questionnaire for data collection. Female investigators in our research team interviewed the participants and completed the questionnaires. They were trained according to ethical and security recommendations for domestic violence researchers [18].

The questionnaire was designed based on a WHO multicountry study and study in India [19, 20]. The questionnaire covered demographic details and experiences of domestic violence at some point in life.

We entered data through* SPSS* (version 16) and analyzed it using* Minitab* and* SPSS*. Chi-squared test and binary logistic regression were carried out.

We asked questions about specific behaviours related to physical violence (beating, hitting with an object, physical injuries, fractures, and hospitalization), psychological violence (verbal insults, the threat of divorce, the threat of remarriage, forcing the woman to leave the house, and refusing verbal communication with their wife), and sexual violence (unwanted or forced sex). The experience of at least 1 form of these 3 forms of violence was considered a form of violence.

## 3. Results

The participants comprised 600 women from Ahvaz (capital of Khuzestan Province), 400 from Andimeshk, 220 from Abadan, and 600 from Dezful; 29.5% (525) of respondents were of Arab ethnicity and 70.5% (1252) were from the Fars and Lur ethnic groups.

The mean age of the women was 28.8 (standard deviation 6.1; range 14–56) years. The great majority of the women (84.1%) did not work outside the home. Consanguineous marriage was reported by 41.2% of women and forced marriage was reported by 16.1%. Demographic characteristics of the different ethnic groups are presented in Table 1. The highest level of education for both women and their husbands was reported in Dezful. A minority of the participants, 9.1% of Arab women and 17.7% of non-Arab women, were in employment. Having a college education was reported by 8.2% of Arab and 22.7% of non-Arab participants (*P* < 0.001).

The majority of the Arab participants were resident in Ahvaz (69.7%) and Abadan (22.1%). Because of similarities for many characteristics in the non-Arab ethnic groups (Fars and Lur) we merged the related data for them in the regression model.

The prevalence of physical violence increased with the age of the woman except for age under 18 years: recorded prevalence was 20.8% for women under 18 years, 17.8% for women aged 18–24 years, 19.1% for women aged 25–29 years, 20.6% for women aged 30–34 years, 24.3% for women aged 35–39 years, and 25.25% for women aged 40+ years.

Prevalence of psychological violence and any form of violence was greater in women aged 40 and over. The greater prevalence of sexual violence was reported in the age group of 30–34 years. The lowest prevalence of various forms of violence in the lifetime was reported in women with a university education level. The experience of all forms of violence was greater in women who did not work outside the home and those whose age at marriage is less than 18 years. There was a significant association between physical violence and psychological, sexual, and any form of violence (*P* < 0.001).

History of psychiatric disorder in husband was related to the experience of all forms of violence in women (*P* < 0.01).

The prevalence of physical, psychological, and any form of violence in the lifetime was the highest in Dezful (25.7%, 54.8%, and 57.7%, resp.). The greatest experience of sexual violence was reported in Ahvaz (17.7%). Overall, Abadan had the lowest prevalence for each form of violence among the 4 cities (Table 2).

The prevalence of physical, psychological, sexual, and any form of violence in the Fars ethnic group was 21.1%, 43.3%, 8.7%, and 47.3% compared to 14%, 46.9%, 10.1%, and 51.5% in the Lur population, respectively. Except for physical violence, there was no statistically significant difference between the Fars and Lur ethnic groups for all forms of violence.

The highest prevalence of physical and sexual violence at any point of life was reported by Arab women (25.1% and 16.7%). Psychological violence and any form of violence were the highest in the non-Arab ethnic group (44.4% and 48.6%, resp.) (Table 3).

The experience of any form of violence was significantly associated with city of residence and ethnicity after adjusting for confounding variables. Results of unadjusted odds ratio are presented in Table 4.

## 4. Discussion

Experience of any form of violence during their lifetime for the women in our study was 47.3%. The highest prevalence of domestic violence in this study is comparable to the findings of other studies from around the world. Estimates of the prevalence of this phenomenon, however, vary widely owing to differences in study populations, definitions of violence, and sociocultural factors [1, 21].

The experience of any form of violence was significantly associated with age of the woman, education and employment of women, history of psychiatric disorder in husband, city of residence, and ethnicity. Some demographic characteristics such as older age of the woman, lower level of education in women and their husbands, not being in employment, and psychiatric disorders in husband have been recognized as risk factors for domestic violence in previous studies [1, 22, 23].

The experience of all forms of violence had significant association with city of residence. There have been no similar studies in this area for comparison of results.

Some studies have reported variation in the prevalence of domestic violence between neighboring areas. These local differences are often greater than differences across national boundaries. All of the forms of violence except psychological violence had a significant association with ethnicity, regardless of the education level and occupation situation of the women. Sociocultural factors may explain ethnic differences in violence against women. Studies across many different cultures have shown some societal and cultural variables that might give rise to higher levels of violence against women [24].

Our findings show the pattern of experience of various forms of violence differs between Arab and non-Arab women: physical and sexual violence prevalence was greater in Arab women and psychological violence was greater in the non-Arab ethnic group. Our study showed some associations between the different forms of violence experienced; similarly, other studies suggest a significant overlap between physical, sexual, and psychological violence [25–27]. Accordingly, therefore, we had expected to report a higher prevalence of psychological violence in Arab women. The reasons for this unexpected result may be the different cultural perceptions about a particular behavior as being violent: acceptance of some forms of violence as “normal” and only reporting those forms of violence that victims themselves class as violence/abuse. There are no similar studies in this area and country for comparison of the results.

Improved education of health professionals about signs of physical and psychological violence could be helpful for active screening and timely referral of victims, particularly in Dezful and centres which women of Arab ethnicity attend. Programs for the prevention of domestic violence in health centers should be integrated with other plans where women are targeted, such as sexually transmitted disease prevention, prenatal care, and other services in family health practice. Primary prevention programmes should be focused on increasing access of women to education; preventing all forms of violence; enabling families (education of women and their husbands about outcomes of domestic violence, particularly in women who marry before they are 18 years old, those with little education, and other high risk groups); forming community groups, including religious communities, and changing community norms; reducing acceptance of violence in Arab women; and increasing domestic violence facilities for women who attend health centres in Dezful city.

Our study had some limitations. The study population comprised women who were attending public health centres for contraception and child care in the 4 cities we selected, and they may not be representative of the total female population of these cities; in particular, older women were not represented. Most women, however, come into contact with the health care centres at some point in their lives for obtaining contraception, for instance, or seeking care for their children. This makes the health care setting an important place for the identification, support, and referral, if necessary, to specialized services [27]. Also, the cross-sectional design, analysis, and conclusions based on self-reporting, probability of error in the definition of violence variables, recall bias in reporting of violence throughout the lifetime, and cultural barriers in the design of questions relating to details of sexual violence are some limitations of the present study.

Despite the limitations, this is the first study with a relatively large sample size in the Islamic Republic of Iran to study domestic violence in ethnic groups. Our study can provide a guideline for health care providers with useful information for planning and screening in high-risk women.

## Figures and Tables

**Table 1:** Demographic characteristics of women participants in Khuzestan based on ethnic group.

Characteristic	**Total (** **n** = 1820**)**		Arab (*n* = 525)		Non-Arab (*n* = 1252)				*P* value
					Fars (*n* = 864)		Lur (*n* = 388)	
	Number	%	Number	%	Number	%	Number	%
Age (years)									<0.001
<18	24	1.3	14	2.7	8	0.9	2	0.5	
18–24	538	29.6	191	36.4	221	25.6	108	27.8	
25–29	549	30.2	144	27.4	259	30.0	138	35.6	
30–34	349	19.2	95	18.1	171	19.8	81	20.9	
35–39	206	11.3	61	11.6	108	12.5	35	9.0	
≥40	151	8.3	20	3.8	95	11.0	24	6.2	
Woman's education									<0.001
Illiterate	75	4.7	39	7.5	19	2.2	15	3.9	
Elementary school	257	14.2	147	28.1	36	4.2	74	19.1	
Less than high school	369	20.4	161	30.8	130	15.1	74	19.1	
High school	773	42.7	133	25.4	437	50.6	179	46.3	
College	337	18.6	43	8.2	238	27.5	45	11.6	
Woman's occupation									<0.0001
Housewife	1521	83.6	472	90.9	669	77.5	360	93.0	
Employed	287	15.8	47	9.1	194	22.5	27	7.0	
Woman's age at marriage (years)									<0.001
<18	425	23.4	177	33.8	142	16.4	102	26.3
18–24	1144	63.0	289	55.2	606	70.1	220	56.7
25–29	209	11.5	49	8.8	97	11.2	59	15.2
≥30	39	2.1	12	2.3	20	2.3	7	1.8	
Husband's education									<0.001
Illiterate	60	3.3	29	5.6	13	1.5	17	4.4	
Elementary school	214	11.8	103	19.9	48	5.6	62	16.0	
Less than high school	458	25.2	192	37.1	147	17.0	109	28.1	
High school	651	35.8	139	26.8	359	41.5	136	35.1	
College	428	23.5	55	10.6	297	34.3	64	16.5	
Consanguineous marriage									<0.001
Yes	749	41.2	273	52.0	243	28.1	224	57.7	
No	1071	58.8	252	48.0	621	71.9	164	42.3	

Some values missing.

**Table 2:** Prevalence of violence against women experienced at any time of life in 4 cities in Khuzestan.

City of residence	Type of violence, % (95% confidence interval)^a^			
	Physical	Sexual	Psychological	Any form
Dezful (*n* = 600)	25.7 (22.2–29.4)	8.5 (6.3–11.1)	54.8 (50.7–58.8)	57.7 (53.6–61.7)
Andimeshk (*n* = 400)	14.0 (10.7–17.8)	9.8 (7.0–13.1)	46.5 (19.5–51.5)	51.0 (45.9–56)
Ahvaz (*n* = 600)	24.0 (20.6–27.7)	17.7 (14.7–21.0)	30.2 (26.5–34.0)	41.7 (37.7–45.7)
Abadan (*n* = 220)	8.2 (4.8–12.8)	2.7 (1.0–5.8)	22.7 (17.4–28.8)	27.7 (21.9–34.1)

^a^Using *Minitab*.

**Table 3:** Prevalence of violence experienced at any time of life by ethnic group.

Ethnicity	Type of violence, % (95% confidence interval)^a^			
	Physical	Sexual	Psychological	Any form
Arab (*n* = 525)	25.1 (21.4–29.1)	16.7 (13.5–20.1)	34.5 (30.4–38.7)	46.1 (41.7–47.2)
Non-Arab (*n* = 1252)	18.8 (16.6–21.1)	9.2 (7.6–10.9)	44.4 (41.6–47.2)	48.6 (45.8–51.4)

^a^Using *Minitab*.

**Table 4:** Binary logistic regression for risk factors for domestic violence against women in Khuzestan.

Characteristic	Type of violence			
	Physical	Psychological	Sexual	Any form
	OR (95% CI)	OR (95% CI)	OR (95% CI)	OR (95% CI)
Woman				
Age	0.97∗∗ (0.95–0.99)	0.96∗∗∗ (0.95–0.98)	1.01 (0.99–1.03)	0.97∗∗ (0.96–0.99)
Education	1.14 (0.97–1.34)	1.19∗ (1.04–1.37)	1.48∗∗∗ (1.22–1.8)	1.27∗∗ (1.11–1.45)
Employment	2.45∗∗∗ (1.50–4.01)	1.50∗ (1.08–2.09)	1.31 (0.68–2.29)	1.56∗∗ (1.13–2.15)
Age at marriage	0.99 (0.96–1.03)	1.02 (0.99–1.05)	0.95∗ (0.91–0.99)	1.01 (0.98–1.04)
Ethnicity	2.56∗∗∗ (1.81–3.63)	1.13 (0.85–1.51)	1.83∗∗ (1.22–2.75)	1.40∗ (1.07–1.84)
City of residence	1.82∗∗∗ (1.53–2.16)	1.90∗∗∗ (1.67–2.17)	1.27∗ (1.04–1.56)	1.78∗∗∗ (1.57–2.02)
Husband				
Literacy	1.23∗∗ (1.06–1.42)	1.03 (0.90–1.17)	1.08 (0.90–1.30)	1.01 (0.89–1.14)
History of psychiatric disorder	1.93∗∗ (1.20–3.11)	2.52∗∗∗ (1.62–4.08)	2.27∗∗ (1.32–3.90)	2.07∗∗ (1.30–3.29)

^*^ *P* < 0.05; ^**^ *P* < 0.01; ^***^ *P* < 0.001.

OR: odds ratio; CI: confidence interval.

## References

[1] Flury M., Nyberg E., Riecher-Rössler A. (2010). Domestic violence against women: definitions, epidemiology, risk factors and consequences. *Swiss Medical Weekly*.

[2] (2010). *Preventing Intimate Partner and Sexual Violence against Women: Taking Action and Generating Evidence*.

[3] Garcia-Moreno C., Heise L., Jansen H. A. F. M., Ellsberg M., Watts C. (2005). Violence against women. *Science*.

[4] Campbell J., Jones A. S., Dienemann J. (2002). Intimate partner violence and physical health consequences. *Archives of Internal Medicine*.

[5] Wingood G. M., DiClemente R. J., Raj A. (2000). Adverse consequences of intimate partner abuse among women in non-urban domestic violence shelters. *American Journal of Preventive Medicine*.

[6] Maman S., Campbell J., Sweat M. D., Gielen A. C. (2000). The intersections of HIV and violence: directions for future research and interventions. *Social Science and Medicine*.

[7] Chandrasekaran V., Krupp K., George R., Madhivanan P. (2007). Determinants of domestic violence among women attending an human immunodeficiency virus voluntary counseling and testing center in Bangalore, India. *Indian Journal of Medical Sciences*.

[8] Gahari S. H., Panaghi L., Atef-Vahid M. K., Zarei-Dost E., Mohamadi-Aria A. R. (2006). Psychological health and domestic violence. *Medical Sciences Journal of Gorgan*.

[9] Heise L., Ellsberg M., Gottmoeller M. (2002). A global overview of gender-based violence. *International Journal of Gynecology and Obstetrics*.

[10] (2002). *Violence against Women*.

[11] Nojomi M., Agaee S., Eslami S. (2007). Domestic violence against women attending gynecologic outpatient clinics. *Archives of Iranian Medicine*.

[12] Ghazizadeh A. (2005). Domestic violence: a cross-sectional study in an Iranian city. *Eastern Mediterranean Health Journal*.

[13] Nouhjah S., Latifi S. M., Haghighi M. (2011). Prevalence of domestic violence and its related factors in women referred to health centers in Khuzestan province. *Behbood Journal*.

[14] Jahanfar S., Kamarudin E. B., Sarpin M. A. B., Zakaria N. B., Abdul Rahman R. B., Samsuddin R. D. B. (2007). The prevalence of domestic violence against pregnant women in Perak, Malaysia. *Archives of Iranian Medicine*.

[15] Yosefi A. (2002). Social classification of ethnic groups in Iran. *National Studies Journal*.

[16] Baktiary A., Omidbaksh N. (2003). Comparison of background and complication of domestic violence in Babol, 2001. *Behbood Journal*.

[17] Lwange S. K., Lemeshow S. (1991). *Sample Size Estimation in Health Studies: A Practical Manual*.

[18] (2001). *Putting Women First: Ethical and Safety Recommendations for Research on Domestic Violence against Women*.

[19] Babu B. V., Kar S. K. (2009). Domestic violence against women in eastern India: a population-based study on prevalence and related issues. *BMC Public Health*.

[20] Garcia-Moreno C., Henrica A. F. M., Watts C., Ellsberg M., Heise L. (2005). *WHO Multi-Country Study on Women's Health and Domestic Violence against Women. Initial Results on Prevalence, Health Outcomes and Women's Responses*.

[21] Lawoko S., Dalal K., Jiayou L., Jansson B. (2007). Social inequalities in intimate partner violence: a study of women in Kenya. *Violence and Victims*.

[22] Heru A. M., Stuart G. L., Rainey S., Eyre J., Recupero P. R. (2006). Prevalence and severity of intimate partner violence and associations with family functioning and alcohol abuse in psychiatric inpatients with suicidal intent. *Journal of Clinical Psychiatry*.

[23] Howard L. M., Trevillion K., Khalifeh H., Woodall A., Agnew-Davies R., Feder G. (2010). Domestic violence and severe psychiatric disorders: prevalence and interventions. *Psychological Medicine*.

[24] Fernández M. (2006). Cultural beliefs and domestic violence. *Annals of the New York Academy of Sciences*.

[25] Koss M. P., Goodman L. A., Browne A., Fitzgerald L. F., Keita G. P., Russo N. F. (1994). *No Safe Haven: Male Violence against Women at Home, at Work, and in the Community*.

[26] Yoshihama M., Sorenson S. B. (1994). Physical, sexual, and emotional abuse by male intimates: experiences of women in Japan. *Violence and Victims*.

[27] (2002). *World Report on Violence and Health*.

